# Anti-osteoporotic effects and good biocompatibility of novel bioactive carbon quantum dots *in vitro* and in ovariectomized mice

**DOI:** 10.1016/j.isci.2024.111700

**Published:** 2025-01-20

**Authors:** Talante Juma, Guang-Hua Liang, Yang Jiao, Yuan-Yuan Ma, Bing-Xiao Yu, Yi-Min Guo, Xin Yang, Heng Liu, Zhi-Chao Meng, Rui Wang, Hao Wu, Li-Ping Pan, Hao Wang, Ya-Hong Wang, Yong-Ping Cao, Tao Zhang

**Affiliations:** 1 Orthopaedic Department, Peking University First Hospital, Beijing 100034, China; 2 Animal Experiment Center, Peking University First Hospital, Beijing 100034, China; 3 ChinaSchool of Materials Science and Engineering, Beihang University, Beijing 100191, China; 4 Harbin Chengcheng Institute for Material and Life, Harbin 150500, China

**Keywords:** health sciences, chemistry, Physics

## Abstract

Osteoporosis is a prevalent condition among the elderly, and current treatments are limited by their side effects. This study aimed to develop a safe nanocarbon material with anti-osteoporotic properties. A promising candidate, carbon quantum dots (CQDs), was synthesized using a single-step liquid-phase pulse method and characterized by transmission electron microscopy (TEM). To evaluate the biocompatibility and anti-osteoporotic effects of CQDs, they were administered at a dose of 276 μg/mL or a placebo to an osteoporotic mouse model (*n* = 16) for 3 months. Biocompatibility was assessed through monitoring weight changes, general health, blood tests, and H&E staining of visceral organs. To assess bone quality, imaging, histological analysis, and biomechanical tests were performed. The results showed that CQDs significantly inhibited osteoclastic activity, leading to improved bone mass and mechanical strength without obvious toxicity. These findings suggest CQDs as a promising candidate for safer osteoporosis therapies.

## Highlights

• CQDs selectively inhibit osteoclast activity, enhancing bone mass and mechanical strength
• CQDs exhibit excellent biocompatibility *in vivo* with no observed adverse effects
• CQDs present a promising, safer alternative for osteoporosis treatment
• Preliminary data suggest CQDs may offer therapeutic benefits for liver and renal function

## Graphical abstract

**Figure:**
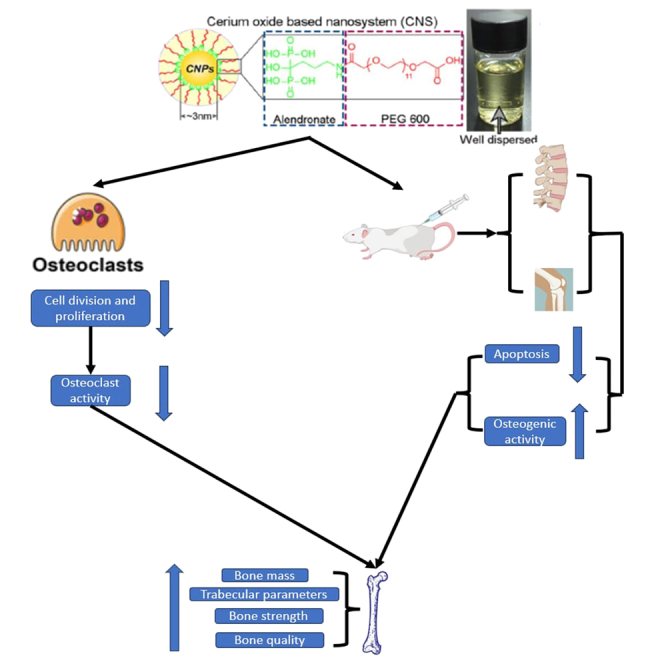

## Introduction

Osteoporosis is a globally concerning systematic disease affecting more than 200 million patients worldwide^1^ and is characterized by low bone mass, a reduction in bone strength, and high susceptibility to fracture. Osteoporotic fracture is very common in elderly individuals and not only increases economic burden but also reduces quality of life and threatens life.^2^ Postmenopausal osteoporosis is the most common type of osteoporosis and manifests as high bone turnover with enhanced bone formation and even stronger bone resorption, which eventually leads to bone loss. Osteoclasts are the only bone-resorbing cells in mammals and predominates bone resorption in postmenopausal osteoporosis patients.^3^ Bone mass can be improved by reducing bone resorption and/or enhancing bone formation. Following this strategy, several drugs have been developed that target osteoclasts and/or osteoblasts, including estrogen, bisphosphonates, and teriparatide. However, all of these drugs are limited in use because of severe side effects or parenteral modes of drug administration^4^; therefore, there is a pressing need to explore new types of anti-osteoporotic drugs.

Nanocarbon materials have been the most studied materials recently due to their unique physical and chemical properties: high stability, high cell permeability, and high functionalization capacity.^5^ Due to their unique properties, carbon quantum dots (CQDs) are emerging as promising candidates for osteoporosis treatment due to their unique properties. These zero-dimensional fluorescent nanomaterials, with a particle size typically less than 10 nm, exhibit a high surface area-to-volume ratio and versatile photoluminescence. Due to their quasispherical morphology and ability to modify their chemical structure—by doping with elements such as nitrogen and phosphorus—both their optical characteristics and biocompatibility are enhanced, which makes CQDs, particularly suitable for biomedical applications, including targeted drug delivery and imaging in osteoporosis therapy. The low toxicity and high solubility of these compounds further support their potential for *in vivo* use.^6^^,^^7^ Despite these advantages, the application of CQDs in osteoporosis is still in its developmental phase. Most current research focuses on environmental and sensing applications, such as UV absorption and formaldehyde detection, rather than therapeutic uses.^6^^,^^7^^,^^8^ A significant challenge lies in optimizing CQDs for specific medical applications, including understanding their biological interactions and ensuring their effectiveness in therapeutic contexts. Additionally, scaling up the synthesis of CQDs from renewable sources, such as biomass, poses practical difficulties that need to be addressed.^6^^,^^7^

The currently studied nanocarbon materials include fullerenes, carbon nanotubes, graphene/graphene oxides, and nanodiamonds. They are considered potential alternatives for various medical applications, such as biosensing, tissue engineering, clinical imaging, photothermal therapy for cancer and drug delivery systems.^9^^,^^10^^,^^11^ Nanocarbon materials in the orthopedic field, which are focused mainly on bone defect repair, are often used as osteogenic agent-bearing scaffolds, but their application in osteoporosis treatment has rarely been reported.^12^ Among these nanocarbon materials, graphene is the most valuable and widely studied material. However, almost all nanocarbon materials, including graphene, are cytotoxic in a time-, size- and dose-dependent manner.^13^

As a promising new class of materials, nanocarbon materials exhibit a wide variety of allotropes with diverse functions, many of which remain undiscovered. Surface modifications can be employed to maximize their biocompatibility. With the unique features mentioned previously, it is possible to develop a new type of nanocarbon material that has excellent bioactivity and biocompatibility. Previously, we reported the synthesis, biocompatibility, and antitumor effects of a new nanocarbon material named CQDs.^14^ This study investigated the effects of CQDs in the orthopedic field, demonstrating their promising potential for the treatment of osteoporosis. The preparation of CQDs is quite simple, only requiring a single-step liquid-phase pulse method, which enables the CQDs to be equipped with many hydroxyl groups.^14^ The mean particle size of the CQDs was approximately 7.5 nm, and these particles formed an amorphous quasispherical microstructure. This study examined the anti-osteoporotic properties of CQDs in ovariectomized mice through imaging, histological, biomechanical, and serological evaluations. This study aims to offer new insights into osteoporosis treatment rather than introducing a new drug.

## Results

### CQDs have a stable nanostructure

**Figure 1:**
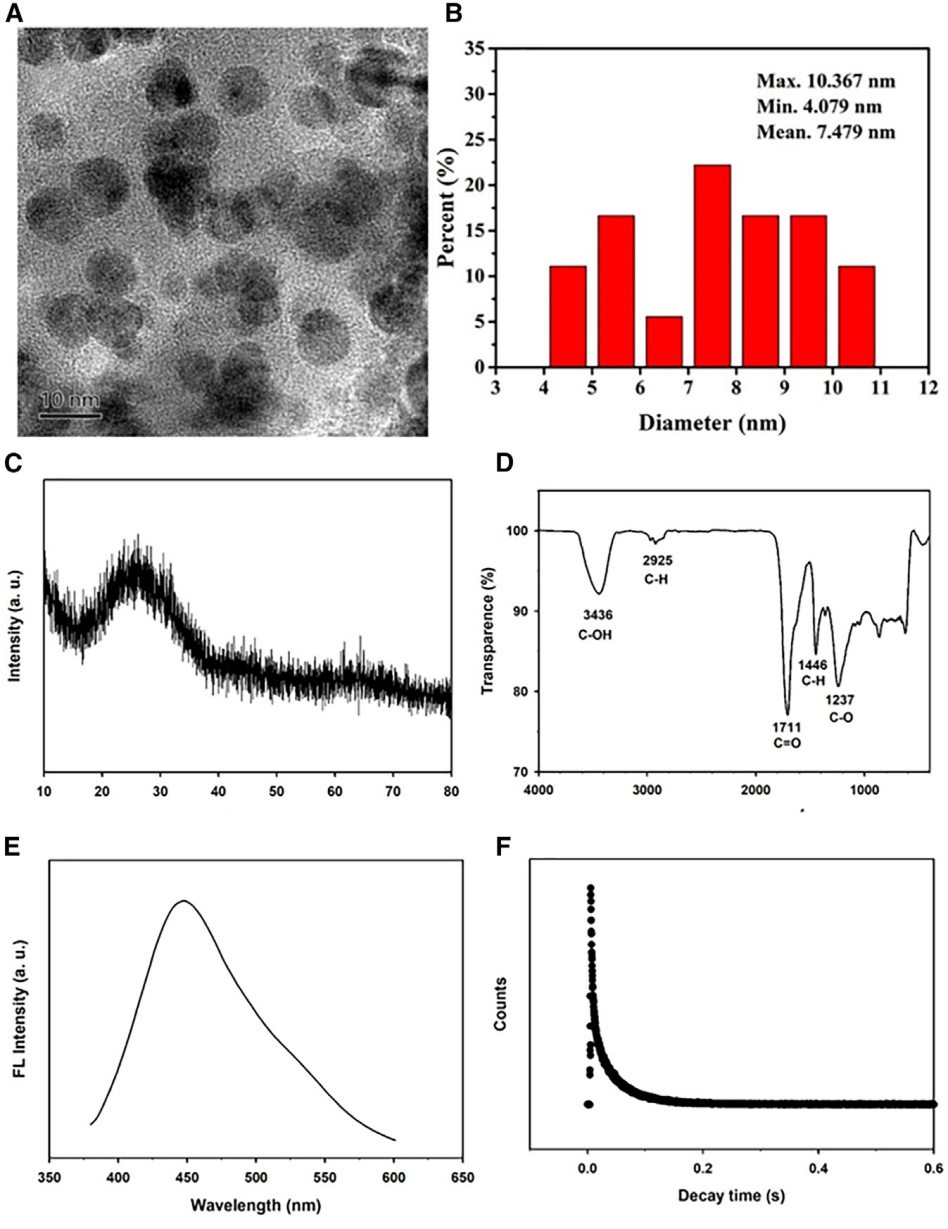
The basic characterizations of the CQDs (A) the TEM images of the CQDs dispersed in the water, (B) the size distribution of the CQDs measured by ImageJ, (C) the XRD pattern of the CQDs, (D) the FT-IR spectrum of the CQDs, (E) the photoluminescence spectra (325 nm) of the CQDs, (F) the time resolved decay spectra of the CQDs.

The characteristics of the CQDs were analyzed using transmission electron microscopy (TEM). Figure 1A shows CQDs have a nearly spherical shape and can be well dispersed in water. Figure 1B shows the size distribution of the CQDs with diameters in the range of 4.079–10.367 nm and an average diameter of 7.479 nm. Notably, the CQDs can be easily dispersed in an aqueous system, suggesting the hydrophilic behavior of the CQDs. To investigate the crystalline structure of the as-prepared CQDs, X-ray diffraction (XRD) was performed. As shown in Figure 1C, the XRD pattern of the CQDs exhibits a broad diffraction peak, which can be attributed to their small particle size. The broad peak suggests that the CQDs possess an amorphous or partially crystalline structure, a characteristic often observed in carbon-based nanomaterials. Fourier transform infrared spectroscopy was carried out to identify the functional groups on the surface of the CQDs (Figure 1D). The broad peak at approximately 3436 cm-1 corresponds to the stretching vibrations of C–OH. The stretching vibrations of C–H (2925 cm-1) and C=O (1711 cm-1) suggest the existence of alkyl functional groups. The absorption peak at 1237 cm-1 indicates the existence of a C–O functional group.^6^^,^^15^^,^^16^ Fourier transform infrared spectroscopy revealed many oxygen-containing functional groups on the surface of the CQDs, which may be vital for the antitumor effects of the CQDs. The photoluminescence properties of the as-prepared CQDs were investigated to evaluate their fluorescence behavior. As shown in Figure 1E, the photoluminescence spectra reveal that the CQDs exhibit strong fluorescence, indicating their potential application in bioimaging and other fluorescence-based technologies.

Furthermore, fluorescence lifetime measurements were conducted to assess the decay dynamics of the CQDs. As shown in Figure 1F, the fluorescence lifetime of the CQDs was determined to be 4.13 ns, suggesting a fast radiative recombination process. This short fluorescence lifetime is typical for carbon-based nanomaterials and further supports the efficient luminescent properties of the CQDs.

### Effect of CQDs on the proliferation and apoptosis of osteoclasts

**Figure 2:**
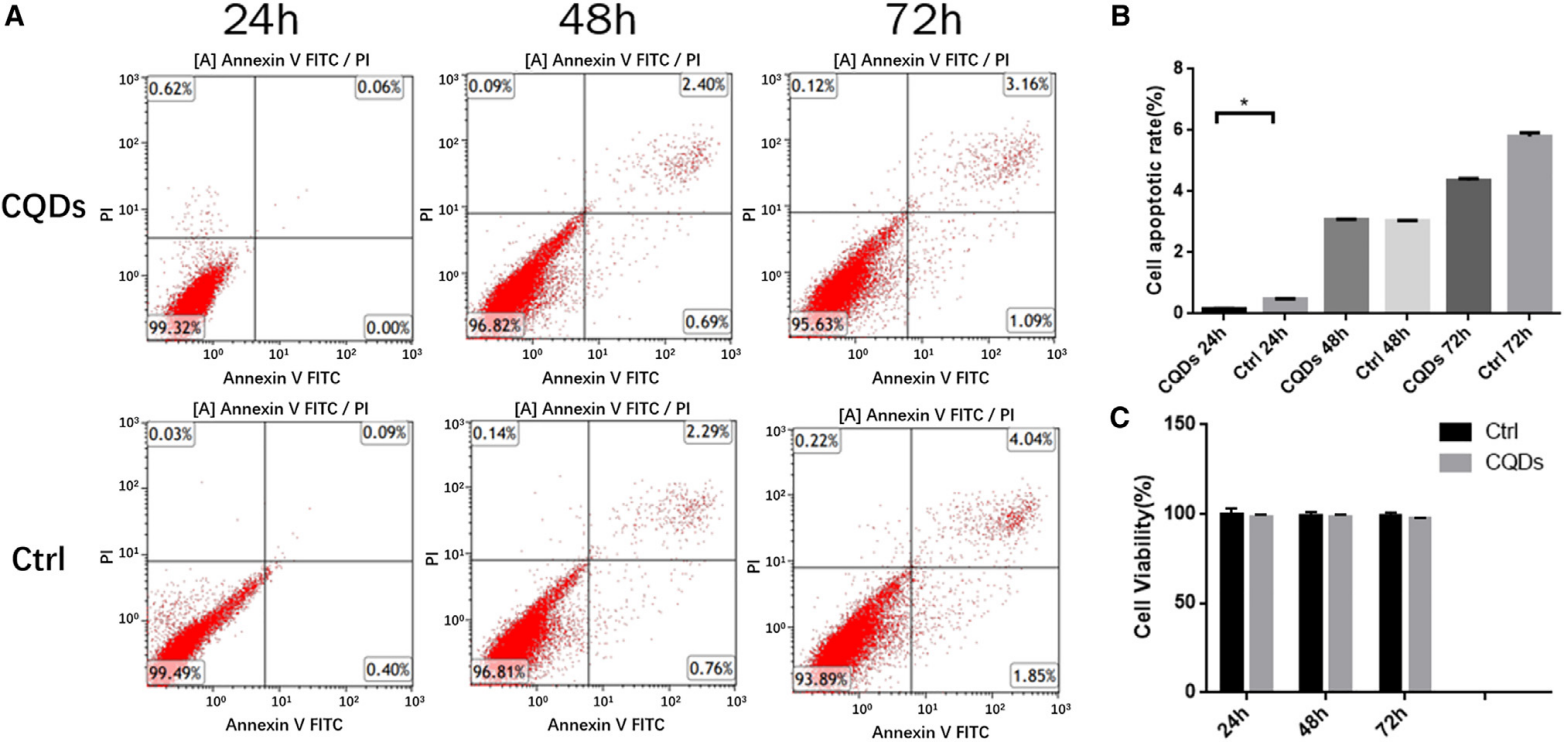
According to the Annexin-V/PI staining analyzed by FCM quantifying early cell apoptotic rate, CQDs did not effect the proliferation and apoptosis of osteoclast (A) The Annexin V/PI staining images of osteoclast co-cultured with CQDs at different time period. (B) Cell apoptotic rate of (A) osteoclast analyzed by FCM. (C) Proliferation levels of osteoclast co-cultured with CQDs by CCK-8 test. Significant differences are marked with ∗(*p* < 0.05, compared to the control).

Osteoclast apoptosis was detected after stimulation with PBS (control group) or CQDs (CQD group) via the Annexin V-fluorescein isothiocyanate method (flow cytometry). The proliferation of osteoclasts cocultured with CQDs was detected via the CCK-8 test. As shown in Figures 2A and 2B, the percentages of apoptotic cells in the Ctrl group at 24 h, 48 h, and 72 h were 0.06%, 3.09%, and 4.25%, respectively, whereas those in the CQD group were 0.49%, 3.05%, and 5.89%, respectively, at the same time points, indicating that there was no significant difference between the two groups. At the level of cell viability, as assessed by the CCK-8 test, there were no significant differences between the CQD group and the Ctrl group at 24 h, 48 h, or 72 h. These results suggest that the CQDs did not affect the proliferation or apoptosis of osteoclasts.

### Effect of CQDs on the cell cycle and the proliferation of osteoclasts

**Figure 3:**
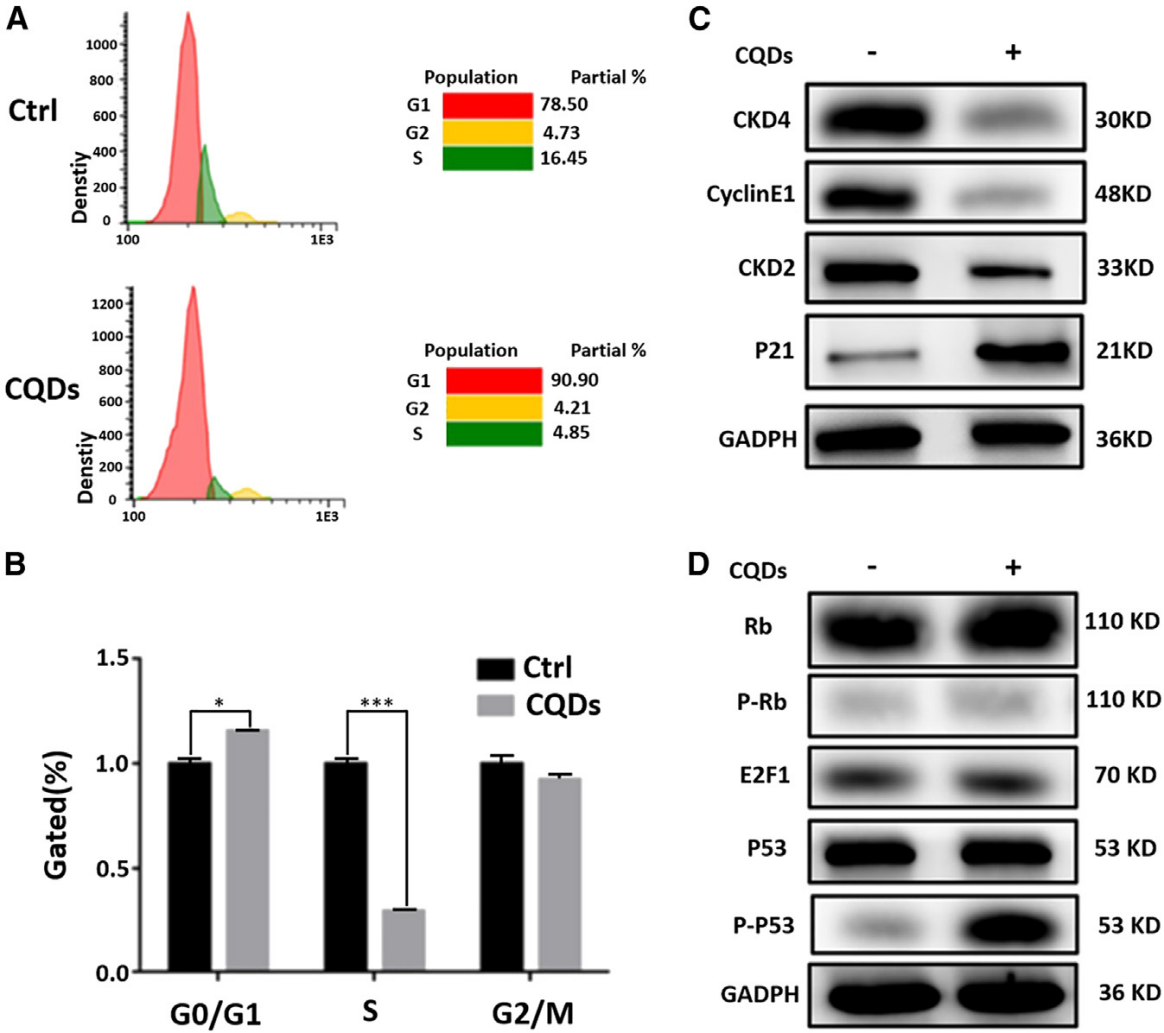
Effects of CQDs on the proliferation of osteoclast cells CQDs decrease the proliferation level of osteoclast. (A and B) FCM analyses of osteoclasts co-cultured with CQDs or PBS. (C and D) Effects of CQDs on the expressions of apoptosis- and cell cycle-related genes in osteoclast. Significant differences are marked with ∗(*p* < 0.05, compared to the control).

To evaluate the impact of the CQDs on the cell cycle of osteoclasts, cell cycle distribution analysis was performed using fluorescence-activated cell sorting on two separate cell groups. As depicted in Figure 3A, osteoclasts co-cultured with CQDs in the experimental group presented a G0/G1 DNA content ratio of 90.90%, whereas in the control group, where the cells were co-cultured with PBS, the G0/G1 DNA content ratio was 78.50%. Moreover, the proportion of cells in the S phase was significantly reduced to 4.85% in osteoclasts co-cultured with CQDs, whereas in the control group, it was 16.45%. In contrast, during the G2/M phase, there was no significant difference in the proportion of cells based on the DNA content between the CQD group (4.21%) and the control group (4.73%). To further investigate the impact of the CQDs on the cell cycle of osteoclasts, western blotting was employed to assess key proteins involved in the cell cycle. As shown in Figures 3C and 3D, the expression levels of crucial proteins that facilitate the progression of the osteoclast cell cycle, including CKD4, cyclin E1, and CKD2, were significantly decreased. In contrast, the expression levels of the cell cycle inhibitory proteins P21, RB, and p-RB, which are known to impede the progression of the cell cycle, were markedly increased. Although there was no significant difference in the protein expression level of E2F1 between the two groups, the expression level of the retinoblastoma (Rb) protein was greater in the CQD group. The Rb protein can inhibit the activity of E2F1 by binding to the transcription factor E2F1, thereby impeding E2F1-mediated gene transcription, particularly of genes associated with DNA synthesis and cell division. The pivotal tumor suppressor gene P53 and its phosphorylated form P-P53, which are typically linked to P53 activity and are implicated in cell-cycle arrest and the induction of apoptosis, were also examined. While there was no apparent difference in the expression levels of P53 between the two groups, P-P53 was notably reduced in the CQD group, as illustrated in Figure 3D. These observations collectively indicate that CQDs inhibited the division and proliferation of osteoclasts.

### The impact of CQDs on the expression of BMP-2, OCN, and TUNEL apoptotic genes *in vivo*

**Figure 4:**
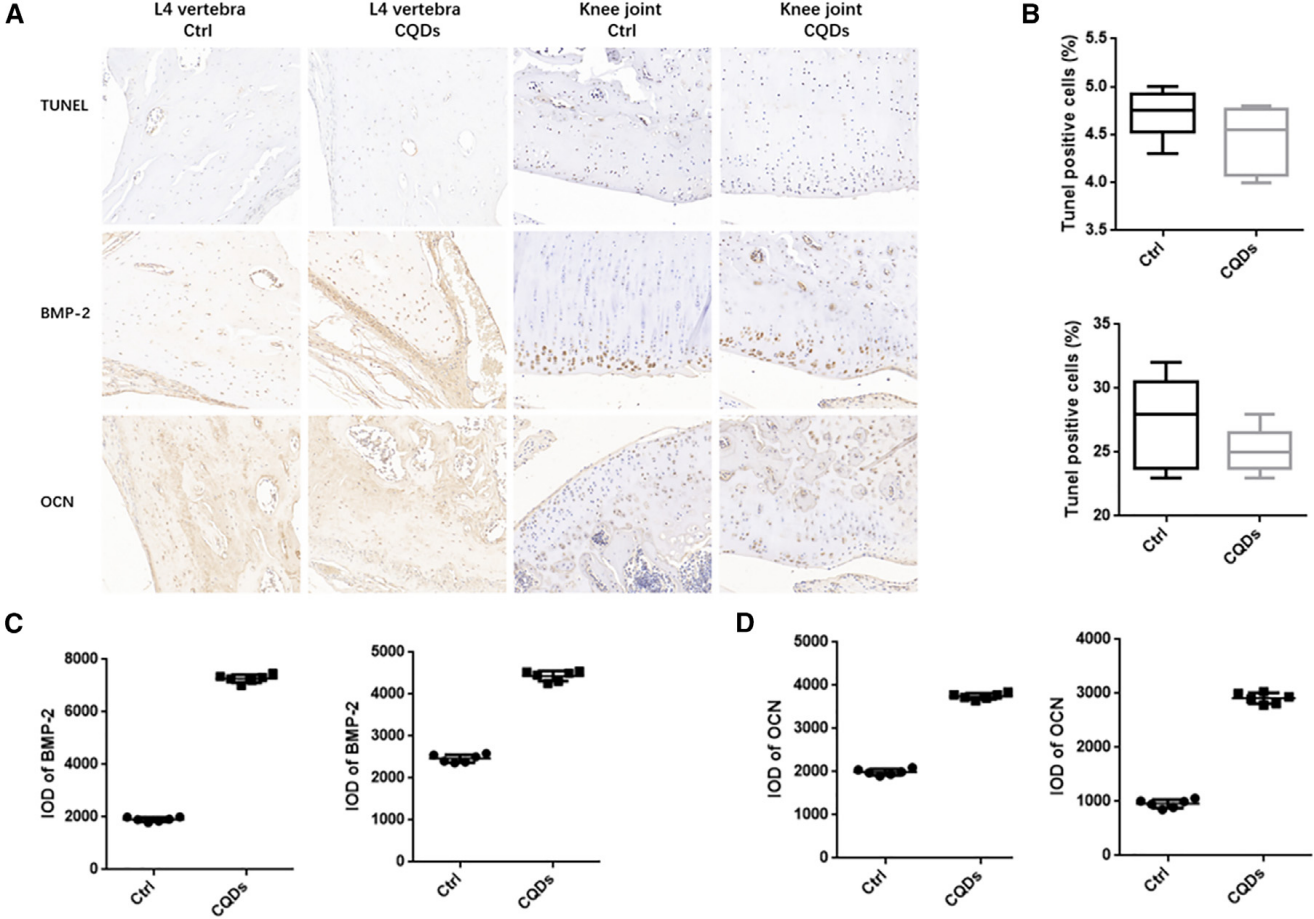
Immunohistochemical analysis of TUNEL, BMP-2, and OCN protein expression in bone tissues of L4 vertebrae and knee joints: a comparison between the control group and the CQDs group (A) The immunohistochemical staining results for TUNEL, BMP-2, and OCN proteins in the L4 vertebrae and knee joints of both the CQDs group and the control group. (B–D) Based on the immunohistochemical results in (A), the IDO values for the expression of TUNEL, BMP-2, and OCN were separately quantified in the CQDs group and the control group. Significant differences are marked with ∗(*p* < 0.05, compared to the control).

The results of immunohistochemistry (Figure 4) indicate that in both the L4 vertebrae and knee joints, the expression of apoptosis-related TUNEL in the CQD group was significantly lower than that in the control group. Conversely, proteins closely associated with osteogenesis, namely, BMP-2 and OCN, exhibited a notable increase in expression in the CQD group. In summary, these findings provide compelling evidence that CQDs significantly enhance osteogenic activity.

### CQDs have excellent biocompatibility

After 12 weeks of treatment, all the BALB/c mice survived, and no significant difference in body weight loss was observed between the two groups (Table 1). There were no differences in reaction, mood or body hair between the two groups during the 12 weeks of drug use. However, the serum biochemistry test results revealed that the levels of ALT, AST, creatinine (Cr), and total bilirubin (Tbil) were notably lower in the CQD group than in the PBS group, while complete blood panel results were not significantly different (Tables 2 and 3). In addition, our previous study on the biocompatibility of CQDs demonstrated that H&E staining of visceral organs, such as the heart, brain, liver, lungs, kidneys, and spleen, revealed no significant histological lesions.^14^

**Table 1:** The body weight, uterine weight, and compression test parameters among groups

Parameters	OVX+PBS		OVX+CQDs	
	Mean	SEM	Mean	SEM
Initial body weight (g)	20.6	1.56	20.8	1.28
Final body weight (g)	31.6	1.87	31.2	1.33
Uteri (g)	0.0327	0.00182	0.0331	0.00151
Fmax (N)	66.9	3.10	92.1∗	4.29
yL (N)	66.0	3.04	91.2∗	4.18
Stiffness (N/mm)	115	3.91	126∗	4.14

Body weight changes at the beginning and end of drug administration, as well as comparisons of L5 vertebral compression test parameters, showed no significant differences in body weight between the two groups during the treatment. However, biomechanical parameters were significantly improved in the CQDs group compared to the PBS group. Significant differences are indicated by ∗(*p* < 0.05 compared to the control).

**Table 2:** Parameters of complete blood test

Items	Complete blood panel test (0 weeks)				Complete blood panel test (12 weeks)				Reference range
	OVX+PBS		OVX+CQDs		OVX+PBS		OVX+CQDs		
	Mean	SEM	Mean	SEM	Mean	SEM	Mean	SEM	
WBC (10^9^/L)	2.87	1.32	2.64	1.13	4.71	1.15	4.12	0.92	0.8–6.8
LC (10^9^/L)	2.45	1.25	2.29	1.08	3.86	1.04	3.57	0.89	0.7–5.7
MONO (10^9^/L)	6.74	2.12	5.94	2.07	15.3	2.79	9.6	1.54	0–30
NEUT (10^9^/L)	0.53	0.19	0.37	0.11	0.69	0.28	0.57	0.11	0.1–1.8
RBC (10^9^/L)	8.74	0.47	8.25	0.51	8.78	0.55	8.33	0.41	6.36–9.42
HB (g/L)	141	4.87	137	3.31	143	5.13	140	4.42	110–143
PLT (10^9^/L)	647	84.2	671	79.5	633	81.2	661	77.6	450–1590

No significant differences were observed in the complete blood panel test parameters.

**Table 3:** Parameters of serum biochemistry test

Items	Serum biochemistry assay (0 weeks)				Serum biochemistry assay (12 weeks)			
	OVX+PBS		OVX+CQDs		OVX+PBS		OVX+CQDs	
	Mean	SEM	Mean	SEM	Mean	SEM	Mean	SEM
ALT	64.1	11.4	62.8	9.63	65.9	9.53	33.4∗	8.29
AST	181	19.5	183	17.4	174	21.7	108∗	18.6
ALP	232	14.9	229	12.8	237	13.8	228	11.5
Cr	105	12.1	106	10.8	99.1	12.1	61.6∗	7.85
BUN	20.2	2.11	18.9	1.43	22.9	1.95	21.4	1.83
TBIL	30.6	4.43	31.2	3.99	39.7	4.43	29.0∗	3.99
TG	1.72	0.189	1.96	0.217	2.24	0.339	1.85	0.287
ALB	32.8	0.79	33.5	0.81	33.7	1.45	34.6	1.61

The levels of ALT, AST, Cr, and Tbil in the CQDs group were notably lower than those in the PBS group. Significant differences are indicated with ∗(*p* < 0.05, compared to the control).

### CQDs enhanced bone mass, trabecular parameters, and bone strength in OVX mice

**Figure 5:**
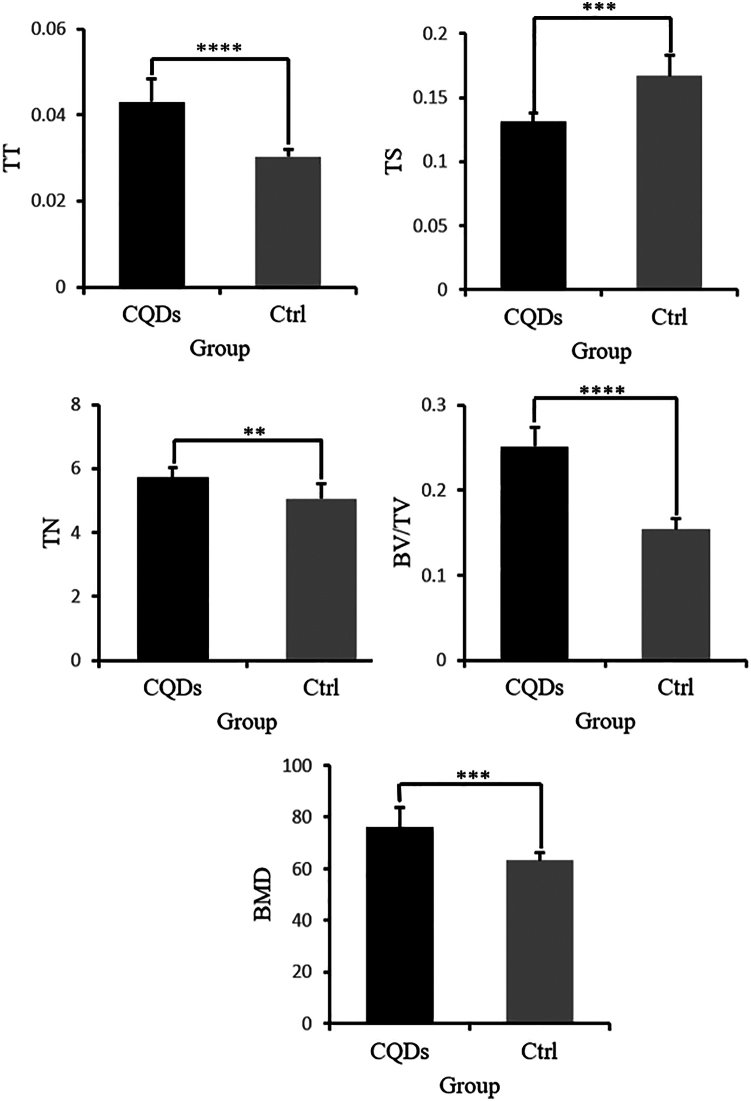
Comparison of bone mineral density (BMD) and trabecular parameters of the L5 vertebrae in mice between the two groups revealed that the BMD, bone volume to total volume ratio (BV/TV), trabecular thickness (TT), and trabecular number (TN) values were significantly higher in the CQDs group compared to the PBS group, whereas trabecular separation (TS) was significantly lower in the CQDs group

**Figure 6:**
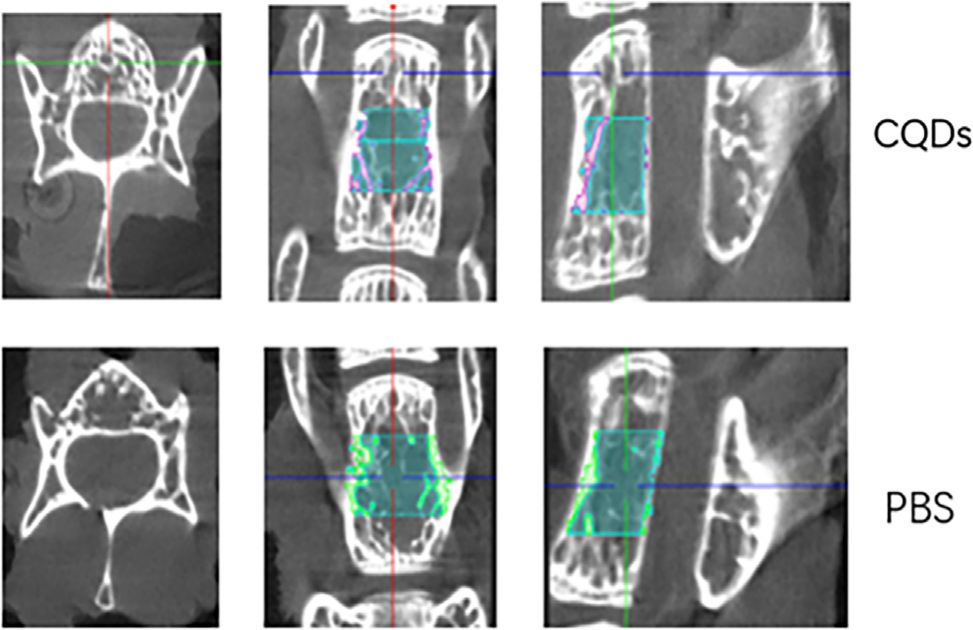
Micro-CT sectional views of the region of interest (ROI) in the L5 vertebra showed a significant increase in cancellous bone in the CQDs group compared to the PBS group across all sectional views

**Figure 7:**
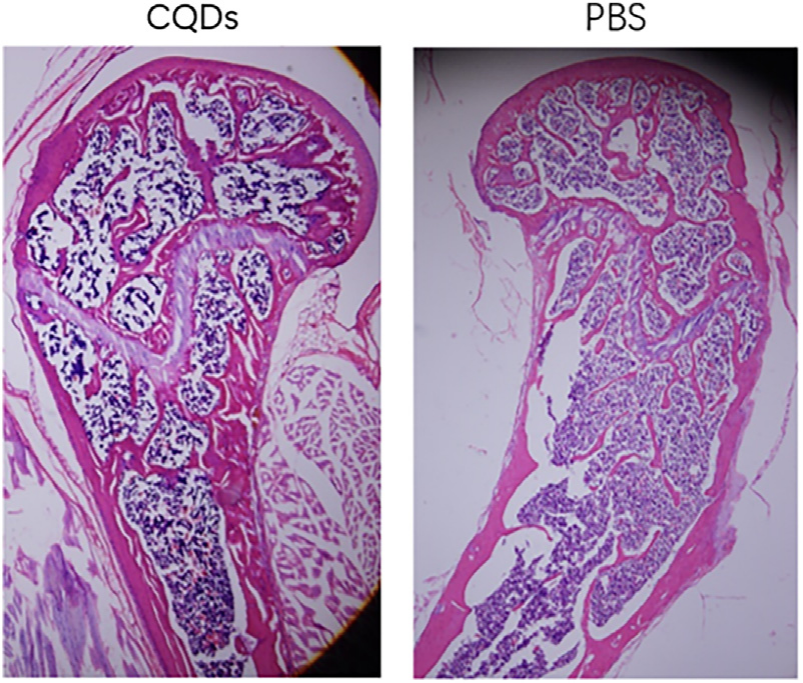
HE staining of femurs in two groups There were more trabecular bone and thicker cortical bone in CQDs group than PBS group. Pictures were captured at 40× magnification.

An OVX-induced mouse osteoporosis model was established to investigate the effects of CQD treatment on osteoporosis, with successful induction confirmed by marked atrophy of the uterus (Table 1). To evaluate bone mass and trabecular parameters, DXA and micro-CT analyses were performed. The mean values of BMD, BV/TV, Tb.Th, and Tb.N in the CQD group were significantly greater than those in the PBS group, whereas Tb.Sp was notably lower in the CQD group (Figure 5). Additionally, micro-CT sectional views of the L5 vertebra and H&E staining of the femur revealed increased trabecular and cortical bone thickness in the CQD group compared with that in the PBS group, indicating an improvement in bone mass with CQD treatment (Figures 6 and 7). To evaluate the bone strength, an L5 axial compression test was performed using a universal testing machine. As shown in Table 1, the maximum load (Fmax), yL and stiffness of the CQD group (92.1 ± 4.29 N, 91.2 ± 4.18 N, and 126 ± 4.41 N/mm, respectively) were significantly greater than those of the PBS group (66.9 ± 3.10 N, 66.0 ± 3.04 N, and 126 ± 4.41 N/mm, respectively). This finding indicated the enhancement of bone mechanical strength and further verified the beneficial effect of the CQDs on bone quality.

### CQDs improved bone quality by suppressing osteoclast activity

**Figure 8:**
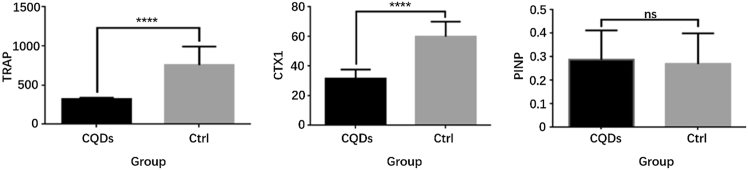
Comparisons of serum bone turnover marker values in two groups There were dramatic decrease of TRAP and CTX1 levels in CQDs group compare to PBS group, which indicated the anti-osteoclastic property of CQDs.

**Figure 9:**
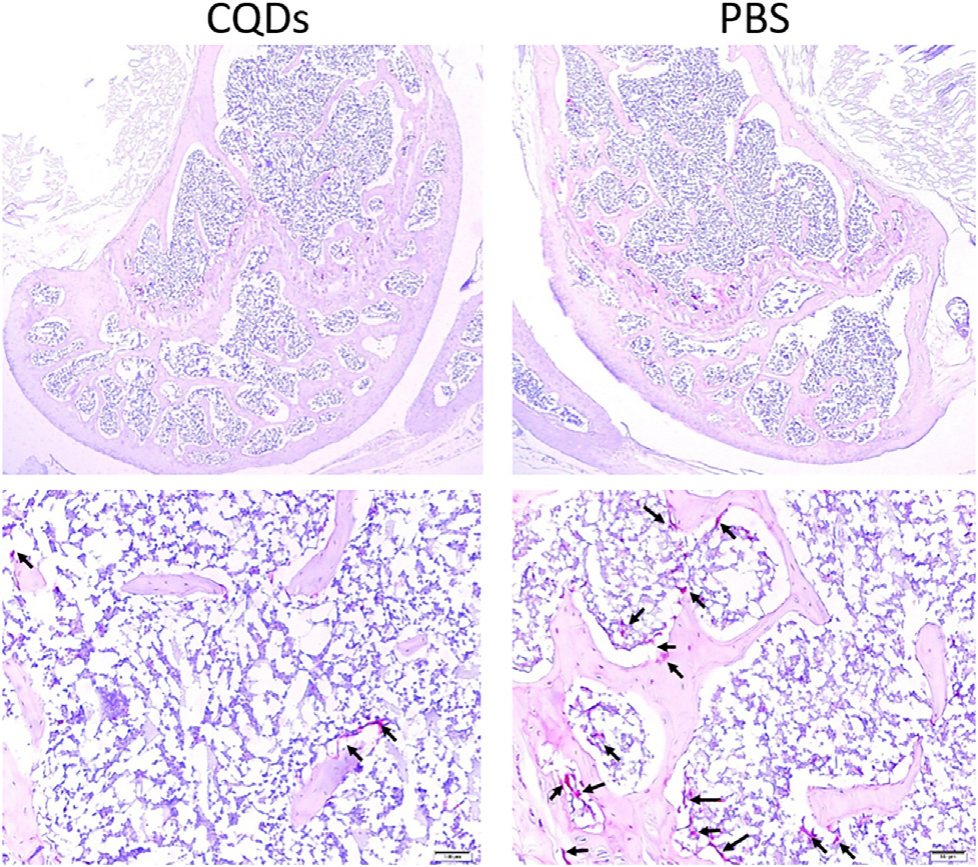
TRAP staining of CQDs and PBS group mice femurs There was significant decrease of TRAP positive cells in CQDs group compared to PBS group. Black arrowheads indicate the TRAP positive osteoclasts, pictures were captured at 40× and 200× magnifications, respectively.

**Figure 10:**
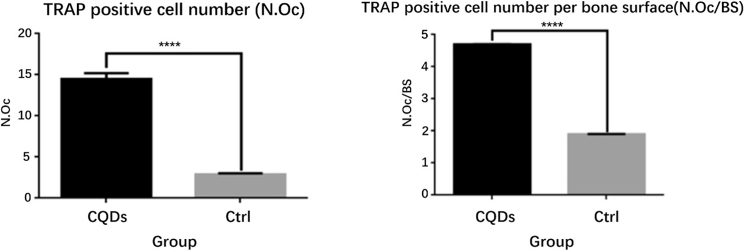
N.Oc and N.Oc/BS in tow groups N.Oc and N.Oc/BS were both significantly decreased in CQDs group compared to PBS group.

To study the preliminary mechanism of the anti-osteoporotic feature of the CQDs, serum bone turnover markers were measured after 12 weeks of drug administration. The serum levels of CTX1 and TRAP in the CQD group were drastically lower than those in the PBS group, while there was no significant difference in the PINP level between the two groups (Figure 8). This finding indicated that bone resorption was lower in the CQD group than in the PBS group and was further confirmed by TRAP staining of femur sections, which revealed that the number of TRAP-positive multinuclear osteoclasts was drastically lower in the CQD group than in the PBS group (Figure 9). The number of TRAP-positive osteoclasts (N.Oc) and the number of TRAP-positive osteoclasts per bone surface (N.Oc/BS) were measured via Bioquant Osteo software, which revealed a remarkable decrease in the N.Oc and N.Oc/BS in the CQD group compared with those in the PBS group (Figure 10).

## Discussion

Two important regulatory factors, receptor activator of nuclear factor κB ligand (RANKL) and macrophage-colony stimulating factor (M-CSF), are necessary for OC differentiation and survival.^2^^,^^3^ The binding of RANKL to its ligand results in the initiation of TNF receptor-associated factor (TRAF) 6 signaling, which eventually activates nuclear factor of activated T cells c1 (NFATc1), the master regulator of osteoclastogenesis,^4^^,^^5^ leading to OC differentiation and maturation.^9^^,^^10^ In the human body, OC has a relatively short cell life of approximately 2 weeks. During this period, OCs differentiate from mononuclear preosteoclasts (pOCs) to multinuclear mature OCs (mOCs) and eventually undergo apoptosis.^11^ Dysregulation of osteoclastogenesis leads to various bone disorders, such as tumor bone metastasis and osteoporosis.^12^^,^^13^^,^^14^

The International Osteoporosis Foundation reported that more than 200 million people suffer from osteoporosis worldwide.^17^^,^^18^ According to a review of the American Society for Bone and Mineral Research, long-term use (over 5 years) of bisphosphonates (BPs), the most commonly used medications for osteoporosis, can lead to unsatisfactory clinical outcomes and complications, including atypical fractures and decreased bone strength.^19^ The detailed reasons for this side effect of BPs are still unclear, but we recently reported that alendronate, a nitrogen-containing bisphosphonate, triggers cell death in pOC through peroxisome dysfunction, and further, the endoplasm barely resorbs the bone matrix; in contrast, they are anabolic for angiogenesis via the secretion of platelet-derived growth factor-BB.^20^^,^^21^^,^^22^ This unselective inhibition of BPs in pOCs and mOCs potentially explains why long-term antiresorptive therapy is not satisfactory. It was hypothesized that the specific depletion of mOCs while preserving beneficial pOCs represents a more effective anabolic strategy for treating osteoporosis and addressing endoplasmic reticulum stress.^23^

Bone metabolism is a dynamic process that requires a balance between bone formation and bone resorption, which is maintained by mechanical stress, hormones, and cytokines.^18^ Osteoporosis is a metabolic disorder associated with systemic bone degeneration, which is caused by aberrant bone resorption. However, abnormal bone resorption is a result of overactivated proliferation of osteoclasts.^19^ For treating osteoporosis, the inhibition of osteoclast formation has been one of the main targets and strategies, but current treatments are clinically limited in terms of side effects, such as osteonecrosis, fever, gastrointestinal reactions, cardiovascular symptoms and increased risk of cancer.^20^^,^^23^ To address the shortcomings of current anti-osteoporosis drug, researchers are focused on developing new drugs with minimal side effects and strong anti-osteoporotic features.

Nevertheless, the modifiable physical and chemical properties of nanocarbon materials, their simple synthesis techniques and their high functionalization compatibility with various ligands and biomolecules have made them highly useful for medical applications, including bioimaging, biosensing, cancer therapy, and drug delivery.^9^^,^^10^^,^^22^ Nanocarbon materials possess an exceptionally high surface area with every single carbon atom exposed at the surface, which facilitates functionalization with high efficiency.^24^ In orthopedic fields, nanocarbon materials have been reported to possess superior traits for bone defect repair.^25^ Nonetheless, increasing evidence has demonstrated that the persistent challenge with nanocarbon materials is their imperfectly modified biocompatibilities.^26^ Theoretically, biocompatibility can vary depending on particle size and functionalization; thus, in this study, a new nanocarbon material called CQDs was developed using a single step liquid-phase pulse method, which enables CQDs to be equipped with abundant oxygen-containing functional groups, such as hydroxyl groups.^14^ Various methods for synthesizing CQDs have been developed, each offering distinct advantages and limitations. Common techniques include laser ablation, electrochemical synthesis, chemical oxidation, and bottom-up approaches, such as microwave irradiation, thermal decomposition, and hydrothermal methods. Among these methods, the hydrothermal method is frequently favored for its eco-friendly nature and cost-effectiveness, particularly in the green synthesis of CQDs from natural resources, such as plant leaves, fruit juices, and other biomass.^27^^,^^28^^,^^29^^,^^30^^,^^31^ In this study, however, we utilized a liquid-phase pulse method with graphite plates as the carbon source, which presents several distinct advantages over traditional methods. Unlike the hydrothermal approach, which typically requires organic or natural precursors, the liquid-phase pulse method is more versatile and uses graphite—a solid carbon source—without the need for additional complex processing steps. This not only simplifies precursor preparation but also eliminates the reliance on multi-step procedures and chemical treatments often associated with hydrothermal methods.^32^^,^^33^ Furthermore, while hydrothermal techniques can introduce variability in particle size and heteroatom doping depending on the carbon precursor used, the liquid-phase pulse method offers greater control over particle uniformity and size distribution, which are key factors in determining the optical and biocompatibility properties of CQDs.^32^^,^^34^^,^^35^^,^^36^ A key advantage of our method is the production of smaller and more uniform CQDs. By applying homogenization at 1.5 kbar, the particle size of the synthesized CQDs was significantly reduced, resulting in finer and more consistent particles. Smaller CQDs, particularly those with abundant surface oxygen-containing groups (e.g., hydroxyl groups), offer enhanced functionalization potential, making them ideal for applications in biomedicine, such as bioimaging and targeted drug delivery.^24^^,^^36^ In comparison, hydrothermal methods, while capable of yielding CQDs with high quantum yields, often involve the use of acids or surface passivating agents, complicating the synthesis process and potentially introducing undesirable byproducts.^28^^,^^35^^,^^37^ Moreover, the single-step synthesis process of the liquid-phase pulse method represents a significant improvement over more complex and resource-intensive methods, such as chemical oxidation or hydrothermal synthesis, which may require strong acids, high temperatures, or multiple steps.^32^^,^^33^ This single-step approach not only simplifies the production process but also enhances scalability, making it highly advantageous for larger-scale CQD production without compromising the consistency of the final product. Importantly, this method adheres to green chemistry principles by avoiding toxic chemicals, further promoting a sustainable and environmentally friendly synthesis route.^28^^,^^35^^,^^38^ According to the TEM results, the average size of the CQD particles was 7.5 nm, and the particles formed stable, amorphous, sphere-like structures. After 3 months of CQD administration, none of the mice showed any signs of weight loss or neurobehavioral changes. Additionally, the complete blood panel and serum biochemistry results and visceral organ H&E staining revealed no abnormalities or lesions, which suggest good biocompatibility of the CQDs. Not only was no harmful effect observed during CQD administration, but instead, the CQDs lowered the serum levels of ALT, AST, Tbil, and Cr, which have never been reported in other types of nanocarbon materials, necessitating exploration of their therapeutic effects on the liver, renal function damage, and metabolic disturbances in the future. Although several studies have shown that nanocarbon materials have beneficial effects on improving bone quality, unlike our findings, most of their materials serve as scaffolds of osteogenic factors.^39^^,^^40^

Although the overall impact of the CQDs on the early apoptosis and proliferation levels of osteoclasts was not statistically significant (Figure 2), further investigation through cell cycle experiments revealed a notable ability of the CQDs to inhibit the cell cycle progression of osteoclasts, thereby suppressing their division and proliferation (Figure 3). Moreover, as evidenced by the trend depicted in Figure 2B, with prolonged exposure to the CQDs, an increase in the percentage of apoptotic osteoclasts was observed. These findings suggest a potential time-dependent effect of the CQDs in promoting osteoclast apoptosis, warranting additional experimental validation in subsequent studies.

In this study, a well-accepted osteoporotic mouse model was established by bilateral OVX, and the success of OVX was confirmed by marked atrophy of the uterus.^41^^,^^42^ The anti-osteoporotic effects of the CQDs were demonstrated by imaging and histological and biomechanical evaluations. According to the DXA and micro-CT results, the CQDs improved the BMD and trabecular parameters, such as TB/BV, TN, and TT, which indicated that the CQDs improved the bone microstructure. In addition, the CQDs enhanced bone quality at both the histological and imaging levels as evidenced in H&E staining of femur sections and sectional micro-CT images, which revealed more, thicker trabecular bone and thicker cortical bone in the CQD group than in the PBS group. After confirming the anti-osteoporotic effects of the CQDs, the preliminary mechanism of their effects on bone mass was studied. As mentioned previously, there are two different ways of improving bone mass: one is by enhancing bone formation, and the other is by reducing bone resorption. Therefore, the serum levels of the main bone turnover markers were measured; CTX1 and TRAP are markers of bone resorption, whereas PINP is the main marker of bone formation. As shown in Figure 6, the levels of CTX1 and TRAP were markedly lower in the CQD group than in the PBS group, while there was no significant difference in the PINP levels between the two groups, which suggested that the CQDs might exert anti-osteoporotic effects by reducing bone resorption. Osteoclast activity is correlated with bone resorption. Nevertheless, TRAP staining is one of the most representative methods for evaluating osteoclast activity.^43^ To further confirm the reduction in bone resorption, osteoclast number and activity were evaluated via TRAP staining of mouse femur sections, which revealed fewer TRAP-positive multinuclear osteoclasts in the CQD group than in the PBS group.

The results of the animal experiments revealed a reduction in TRAP-positive osteoclast numbers and improved bone quality, indicating that the CQDs protected against bone loss in OVX mice via the inhibition of osteoclast activity. Although the CQDs interrupted osteoclastogenesis *in vivo*, the deeper mechanisms of the effects of the CQDs on osteoclast formation and function have yet to be determined, which is necessary for future studies. This study provides evidence that CQDs can act as inhibitors of osteoclastogenesis in the treatment of diseases such as osteoporosis.

### Conclusions

In this study, we successfully synthesized and characterized a novel bioactive nanocarbon material, CQDs, and demonstrated their remarkable anti-osteoporotic effects *in vivo* for the first time. Unlike conventional anti-osteoporotic agents, CQDs exhibit excellent biocompatibility with no observed adverse health effects while significantly increasing bone mass and mechanical strength through the selective inhibition of osteoclast activity. This unique mechanism underscores the potential of CQDs as groundbreaking therapeutic options for osteoporosis, overcoming the limitations and side effects associated with current treatments. Moreover, our findings highlight CQDs as a new class of anti-osteoporotic agents with an innovative mode of action. This study also paves the way for further research into the bioactive properties of CQDs, particularly their effects on liver and renal function, where preliminary data suggest potential benefits. Future investigations will focus on elucidating the detailed molecular mechanisms by which CQDs influence bone metabolism and exploring their applications in other metabolic disorders. In summary, CQDs present a highly effective, safe, and innovative approach for treating osteoporosis and hold promising potential as multifunctional nanomaterials with broad biomedical applications.

### Weaknesses and prospects

Despite the successful synthesis of the novel bioactive CQDs and the demonstration of their significant anti-osteoporotic effects in this study, several limitations warrant consideration. First, the precise mechanisms by which CQDs influence osteoclast function and bone metabolism *in vivo* remain to be fully elucidated. Therefore, further research is essential to investigate these molecular mechanisms in greater depth. Additionally, while the mouse model employed in this study proved effective, it may not be broadly applicable across different age groups and sexes, potentially limiting the generalizability of CQD efficacy in larger, more diverse populations. Future investigations should focus on optimizing the synthesis process of CQDs to enhance their functional characteristics and to explore their applications in other metabolic disorders. Moreover, larger-scale preclinical trials are recommended to confirm the anti-osteoporotic effects of CQDs under varying physiological conditions. By addressing these limitations, CQDs could emerge as a new generation of safe and effective therapeutic options, providing innovative strategies for the management of osteoporosis.

## Resource availability

### Lead contact

Further information and requests for resources and reagents should be directed to and will be fulfilled by the lead contact, Yong-Ping Cao (freehorse66@163.com).

### Materials availability

The readers can purchase the chemicals required to remake the materials as described in the text, or obtain the prepared materials from the corresponding author upon reasonable request.

### Data and code availability

• All data reported in this article will be shared by the corresponding author upon reasonable request.
• This article does not report the original code.
• Any additional information required to reanalyze the data reported in this article is available from the lead contact upon request.

## Acknowledgments

We thank Professor Chun-Li Song and Dr Jun-Xiong Zhu for providing us equipment and technical support on micro-CT and Dual X-ray Absorptiometry scanning.

Funding: This research received no external funding.

## Author contributions

Conceptualization: T.J., G.-H.L., Y.-P.C., and T.Z.; methodology,: T.J., G.-H.L., and Y.-P.C.; software: Y.J.; validation: B.-X.Y.; formal analysis: X.Y. and H.W.; investigation: Y.-H.W. and R.W.; resources: Y.-M.G. and L.-P.P.; data curation: Y.-Y.M. and H.W.; visualization: H.L. and Z.-C.M.; writing—original draft preparation: T.J.; writing—review and editing: G.-H.L. and Y.-P.C.; supervision: Y.-P.C and T.Z.; project administration: Y.-P.C.

## Declaration of interests

The authors have declared no conflicts of interest.

## STAR★Methods

### Key resources table

**Table:** 

REAGENT or RESOURCE	SOURCE	IDENTIFIER
**Chemicals, peptides, and recombinant proteins**		

Graphite plates	Beijing Ji’xing Sheng’an Company (JXSHA, Co., Ltd., China)	JXSHA-2020-013
DNase-free RNaseA	Calbiochem, Germany	CAL-BC-4726
RIPA lysis buffer	Beyotime, Jiangsu, China	BYT-RL-1805

**Critical commercial assays**		

chemiluminescence detection kit	Millipore, USA	MLP-CL-1908
Procollagen Type 1 N-Terminal Propeptide (P1NP) ELISA kits	Nanjing Jian Cheng Bioengineering Institute (Nanjing, China)	NJJ-ELISA-P1NP-0522
The Cross-Linked C-Telopeptide of Type I Collagen (CTX1) ELISA kits	Nanjing Jian Cheng Bioengineering Institute (Nanjing, China)	NJJ-ELISA-CTX1-0319
Tartrate-Resistant Acid Phosphatase (TRAP) ELISA kit	Leagene Biotechnology (Beijing, China)	LGB-TRAP-3007
TRAP staining kit	Servicebio Technology Co., Ltd. (Wuhan, China)	SB-TRAP-2023
ApoScreen annexin V kit	Beyotime Biotech, Shanghai, China	BYT-ANV-0405

**Software and algorithms**		

ImageJ	National Inst.Of Health	https://imagej.nih.gov/nih-image/index.html
Bioquant Osteo software	BioQuant image analysis, USA	https://www.bioquant.com/

**Other**		

transmission electron microscopy (TEM)	JEM-2100F, Tokyo, Japan	JEM-2100F-TKY-2109
copper grid	300 mesh; Ted Pella	TP-300G-2020
flow cytometer	BD Influx, NJ, USA	BD-INF-5103
micro-CT	Inveon, Siemens, Erlangen, Germany	INV-SIM-4512

### Experimental model and study participant details

#### Mice

Only mouse participants were used and no new human participants were recruited for this study. All animal experiments were performed in accordance with the principles and procedures of the National Institutes of Health Guide for the Care and Use of Laboratory Animals and were previously approved by the Animal Ethics Committee of Peking University First Hospital (Approval No. J201847). Briefly, twenty 6-week-old C57BL/6 female mice were purchased from Beijing Vital River Laboratory Animal Technology Co. (Beijing, China) and bred at the Peking University First Hospital animal laboratory. After acclimatization for 2 weeks, all the mice were generally anesthetized and subjected to bilateral ovariectomy (OVX). Six weeks after OVX, two mice were divided randomly into two equal groups: the control group (OVX + PBS) and the CQD group (OVX + CQDs). The mice were group-housed in individually ventilated cages under a 12-hour light/dark cycle, with controlled ambient temperature (22°C) and humidity (50%), and had *ad libitum* access to food and water.

#### Cell culture

Osteoclasts from 6-week-old C57BL/6 female mice were cultured in DMEM containing 15% fetal bovine serum with PBS (control group) or CQDs (CQD group) at 37°C in a 5% CO2 atmosphere.

### Method details

#### Preparation and characterization of CQDs

Graphite plates (100 × 30 × 5 mm, 99.9% pure) were used as the source of carbon, and the CQDs were synthesized via a liquid-phase pulse with double graphite electrodes. To obtain finer particles, the original CQD water solution was homogenized at 1.5 kbar fifteen times for each sample. For transmission electron microscopy (TEM) characterization, the CQDs were cast onto a copper grid (300 mesh; Ted Pella). After drying in air, the sample was observed via TEM (JEM-2100F, Tokyo, Japan) operating at 200 kV, and the primary particle size across the diameter was measured using TEM software (ImageJ).

#### Flow cytometry-based annexin V/propidium iodide (PI) staining

Osteoclasts from 6-week-old C57BL/6 female mice were cultured in DMEM containing 15% fetal bovine serum with PBS (control group) or CQDs (CQD group). After 24 hours, 48 hours and 72 hours, the cells in each group were collected, digested and resuspended in cold binding buffer at a concentration of 106 cells/mL. One hundred microliters of the cell suspension was incubated with 10 μL of labeled annexin V on ice for 15 min. The binding buffer and PI solution were added to the cell suspension according to the ApoScreen annexin V kit (Beyotime Biotech, Shanghai, China) instructions. Subsequently, a flow cytometer (BD Influx, NJ, USA) was used to count the number of stained cells.

#### Cell proliferation assay

The assessment of the cell proliferation ability of the osteoclasts was conducted using the CCK8 assay. To perform the CCK8 assay, the cell culture media of the two groups (the control group and the CQD group) were supplemented with CCK-8 reagent at a ratio of 1:10 (Beyotime, Shanghai, China). Cell proliferation was assessed by measuring the absorbance at a wavelength of 450 nm immediately after incubation at 37°C for 2 h.

#### Cell cycle assay

The cells in the different groups were trypsinized (HyClone, USA), washed once with PBS and fixed with 70% ethanol overnight at 4°C. After fixation, the cells were washed once with PBS, resuspended in PBS/0.1% Triton X-100 and incubated with 50 U of DNase-free RNaseA (Calbiochem, Germany) (30 minutes, room temperature). After incubation, the cells were stained with PI (20 mg/mL in PBS, 15 minutes at room temperature). Flow cytometry (FCM) analysis was performed using a flow cytometer (BD Biosciences, USA).

#### Western blotting

The proteins of different groups of cells were extracted with RIPA lysis buffer containing a protease inhibitor cocktail (Beyotime, Jiangsu, China). Sixty micrograms of protein from each group was loaded, and the proteins were transferred onto nitrocellulose (NC) membranes after electrophoresis. Nonspecific protein binding was blocked with 5% nonfat milk in PBS for 2 h at room temperature. The membranes were incubated with anti-CKD4, anti-CyclinE1, anti-CKD2, anti-P21, anti-Rb, anti-P-Rb, anti-E2F1, anti-P53, anti-P-P53, and anti-GAPDH (1:1,000) antibodies overnight at 4°C. The signal was detected via an enhanced chemiluminescence detection kit (Millipore, USA) after incubation with secondary antibodies conjugated with horseradish peroxidase for 1 h. Then, the immunoreactive bands of the proteins were scanned using ChemiDoc XRS (Bio-Rad) and analyzed via ImageJ software.

#### Ovariectomy-induced osteoporosis in mice

To evaluate osteoporosis induced by bilateral ovariectomy, body weight gain and trabecular bone density of both the femoral head and alveolar bone were compared between the ovariectomy (OVX) and sham groups. Eight weeks post-operation, the weight gain of the OVX mice was significantly greater than that of the sham group (Table 1). Osteoclasts are clearly visible in the cancellous bone of both groups (Figure 9, indicated with black arrows). Micro-CT revealed sparser trabecular bone in the femoral head of the OVX group (Figure 6). Moreover, the bone mineral density (BMD) and bone volume/total volume (BV/TV) of the OVX group decreased, and the trabecular separation (Tb. Sp) value increased (Figure 5). Microarchitectural changes in alveolar bone revealed that trabecular parameters (BMD, BV/TV, and trabecular number (Tb.N)) in OVX mice were significantly lower than those in the sham group (Figure 5). All results revealed obvious bone mass loss and trabeculae deterioration in the femoral head and alveolar bone of the mice that underwent bilateral OVX.

#### Animals and experimental procedures

All animal experiments were performed in accordance with the principles and procedures of the National Institutes of Health Guide for the Care and Use of Laboratory Animals and were previously approved by the Animal Ethics Committee of Peking University First Hospital (Approval No. J201847). Briefly, twenty 6-week-old C57BL/6 female mice were purchased from Beijing Vital River Laboratory Animal Technology Co. (Beijing, China) and then divided randomly into two equal groups: the control group (OVX + PBS) and the CQD group (OVX + CQDs). After acclimatization for 2 weeks, all the mice were generally anesthetized and subjected to bilateral OVX. Six weeks after OVX, two mice were randomly selected from each group and euthanized, and their uteri were isolated for weighing and hematoxylin and eosin (H&E) staining to confirm the success of OVX. Afterwards, the remaining 16 mice were given 0.5 mL of PBS or 0.5 mL of CQDs (276 μg/mL) orally once a day and were injected with 0.5 mL of PBS or CQDs (276 μg/mL) intraperitoneally twice a week for 12 weeks. All mice were euthanized at the end of 12 weeks, and the left femur and lumbar spines from each mouse were fixed in 4% paraformaldehyde for histological studies, immunohistochemistry, micro-CT, BMD scanning, and L5 compression tests. Visceral organs were collected for H&E staining, and blood samples were collected for evaluation of complete blood panel, serum biochemistry, and CTX-1, P1NP, and TRAP levels.

#### Biocompatibility evaluation

Each group of mice was weighed at the beginning and end of drug administration, and weight changes were monitored. Body hair, mood and reactions were also monitored throughout the entire study period. Blood samples from all the mice were collected for evaluation of complete blood panel and serum biochemistry and were separated for H&E staining at the end of the study.

#### Micro-CT scanning

Fixed lumbar spine specimens (n = 8 per group) were scanned via micro-CT (Inveon, Siemens, Erlangen, Germany) at an effective pixel size of 8.82 μm, 80 kV/500 μA, and a 1500-ms exposure time in each of the 360 rotational steps. The BV/TV, trabecular thickness (TT), TN, and trabecular separation (TS) of L5 vertebrae were measured via Inveon software. Different sectional views of L5 vertebrae were also captured to compare the bone mass between the two groups.

#### DXA scanning

The BMD of dissected L5 vertebrae (n = 8 per group) from all the mice (uncalcified) was measured via dual X-ray absorptiometry (DXA) (Ultrafocus, Faxitron, USA). The BMD of each vertebra was measured by 1 technician blinded to the specimen treatment group.

#### L5 vertebra compression test

Separately dissected, uncalcified intact L5 vertebrae (n = 8 per group) were subjected to compression tests via a universal testing machine (50 kN, CA, USA) according to a previously published protocol [12]. The loading rate was 0.2 mm/sec with an initial force of 1 N, and the measured range of the mechanical force was from 2 N to 500 N at a relative accuracy of 0.2% – 0.4%. Finally, the load‒displacement curves were used to calculate the maximum load (Fmax), yield load (yL) and stiffness (S). Fmax is the highest force that the bone withstands. The bending point from elastic deformation to plastic deformation is designated as yL. Stiffness measures the elasticity of a bone. Regression analysis was performed with GraphPad Prism 6 software to determine the differences in the parameters of each group. Similarly, the data of each sample were analyzed by 1 technician who was blinded to the specimen treatment group.

#### H&E staining and TRAP staining

The left femurs (n=8 per group) were isolated and fixed in 4% paraformaldehyde for 24 h, decalcified in 10% EDTA for 3 weeks, dehydrated with different concentrations of alcohol and embedded in paraffin. Longitudinal sections with a thickness of 6 μm were cut and stained with H&E or TRAP in accordance with the manufacturers’ protocols. All the samples were examined and photographed under a high-quality microscope. The intensity of red color is proportional to TRAP activity, and the TRAP-positive multinuclear osteoclast number (N.Oc) was calculated via Bioquant Osteo software and normalized to the total bone surface (BS) in 6 independent fields of view for every single slice (BioQuant image analysis, USA).

#### Bone turnover markers

Mouse serum was collected from the extracted eyeball blood and stored at 4°C after being allowed to clot for 30 min at room temperature. The samples were subsequently centrifuged at 3000 × g and 4°C for 10 min to obtain the serum. Serum CTX-I, P1NP and TRAP levels were determined via ELISA kits following the manufacturer’s protocols.

#### Reagents

Graphite plates (100×30×5 mm, 99.9% pure) were purchased from Beijing Ji’xing Sheng’an Company (JXSHA, Co., Ltd., China). The Cross-Linked C-Telopeptide of Type I Collagen (CTX1) and Procollagen Type 1 N-Terminal Propeptide (P1NP) ELISA kits were purchased from Nanjing Jian Cheng Bioengineering Institute (Nanjing, China), the Tartrate-Resistant Acid Phosphatase (TRAP) ELISA kit was purchased from Leagene Biotechnology (Beijing, China), and the TRAP staining kit was purchased from Servicebio Technology Co., Ltd. (Wuhan, China).

### Quantification and statistical analysis

The data are presented as the means ± standard deviations. A paired t test was used to determine the differences in the bone microstructure parameters, BMD, biomechanical strength and serum bone turnover markers between the two groups. For all the statistical tests, p <0.05 indicated a significant difference between the groups.
